# What Are the Ethical Issues Facing Global-Health Trainees Working Overseas? A Multi-Professional Qualitative Study

**DOI:** 10.3390/healthcare4030043

**Published:** 2016-07-13

**Authors:** James D. Harrison, Tea Logar, Phuoc Le, Marcia Glass

**Affiliations:** Division of Hospital Medicine, University of California San Francisco, San Francisco, CA 94143, USA; tea.logar@gmail.com (T.L.); phuoc.le@ucsf.edu (P.L.); marcia.glass@ucsf.edu (M.G.)

**Keywords:** global health education, global health training, ethics, global health electives, healthcare education

## Abstract

The aim of this study was to identify global health ethical issues that health professional trainees may encounter during electives or placements in resource-limited countries. We conducted a qualitative study involving focus groups and an interview at the University of California San Francisco. Participants were multi-professional from the Schools of Medicine, Nursing and Pharmacy and had experience working, or teaching, as providers in resource-limited countries. Eighteen participants provided examples of ethical dilemmas associated with global-health outreach work. Ethical dilemmas fell into four major themes relating to (1) cultural differences (informed consent, truth-telling, autonomy); (2) professional issues (power dynamics, training of local staff, corruption); (3) limited resources (scope of practice, material shortages); (4) personal moral development (dealing with moral distress, establishing a moral compass, humility and self awareness). Three themes (cultural differences, professional issues, limited resources) were grouped under the core category of “external environmental and/or situational issues” that trainees are confronted when overseas. The fourth theme, moral development, refers to the development of a moral compass and the exercise of humility and self-awareness. The study has identified case vignettes that can be used for curriculum content for global-health ethics training.

## 1. Introduction

The last decade has witnessed a steady increase in the number of health science trainees from medicine, nursing and pharmacy traveling to resource-limited countries to engage in global-health activities [1]. This increase is a reflection of the presence of global health electives and training programs within many health sciences curricula [2]. Trainees from the health professions often interact with vulnerable populations and participate in direct patient care, research, capacity building, program development, and policy work. Global health electives have been shown to be valuable, beneficial and highly relevant for those trainees and health professionals who participate [3,4,5]. Benefits include a better understanding of other cultures, an appreciation of global socio-political health issues and the development of cultural competence [4]. Trainees who complete such electives are also more likely to focus their clinical career working with disadvantaged communities and often display increased levels of volunteerism and humanitarianism [3].

Participation in global health electives has the potential to generate unique ethical dilemmas for which trainees and host communities may often be ill-prepared [6]. For example a qualitative study involving 24 medical students who participated in an international learning trip reported a struggle between local and foreign providers in terms of paternalistic versus shared approaches to patient decision-making [7]. A different study, involving Canadian resident psychiatrists self-reflecting on their work in Ethiopia, found that they had entered their placement with a number of pre-existing biases towards their host institutions and communities [5]. Inadequate preparation for distinctive ethical challenges can yield adverse consequences for trainees and their patients, especially because global-health work requires adapting to locality-specific norms that trainees are unlikely to encounter at their home institutions [6]. Historically pre-departure training for global health electives has been highly varied [8] but more recently, in recognition of ethical conflicts facing trainees, there have been increasing efforts to introduce more substantive pre-departure training in global-health ethics [9,10,11]. The foundation of such training can be practice guidelines published by Crump and Sugar [9]. Potential ethical scenarios are often presented as case studies or vignettes. Teaching modalities for this training can be either in-person didactic sessions, online or simulation [12,13,14,15,16]. This study seeks to add to the number and variety of vignettes that are available to include in global health ethics curricula and training. The aim of this study is to identify important ethical issues that trainees could encounter during placements in resource-limited countries.

## 2. Methods

### 2.1. Setting

We conducted a qualitative study at the University of California San Francisco (UCSF) between November 2013 and January 2014. The Institutional Committee on Human Research approved our study.

### 2.2. Sample and Recruitment

We recruited a group of global-health faculty via a snowball-sampling method [17]. In snowball sampling, the research team recruits a few eligible participants who are then asked to identify additional participants who are then contacted and invited to participate in the study. These additional participants are then also asked to identify more participants who are then invited. This approach continues until an adequate number of participants are recruited into the study [18]. To ensure multi-professional input, we initially identified participants from the UCSF Schools of Medicine, Nursing and Pharmacy who were known to have professional experience working, or teaching, as providers in resource-limited settings. We sent an email invitation to potential participants describing the aims of the study, requesting participation and identification of additional potential participants. We then sent the same email invitations to those potential participants until representation from each multi-professional group was confirmed.

### 2.3. Data Collection

We conducted focus groups or an individual interview with faculty members with global-health experience. The variation in the approach to data collection was due to feasibility and practical reasons to enable faculty participation. We held separate focus groups for medicine, nursing, and pharmacy professionals to enable exploration of each profession’s unique perspective. At the beginning of the focus group and interview, we informed participants that the aim of the session was to identify ethical issues that global health trainees may face when placed overseas that then could be used to inform the content of curriculum for global-health ethics pre-departure training. We then asked participants to personally reflect and specifically discuss any actual ethical dilemmas they had experienced, or were aware of, from working in resource-limited settings or teaching global health. The discussion was unstructured allowing participants to address the question in-depth. We audio-recorded all focus groups and the interview.

### 2.4. Data Analysis

We transcribed each of the focus groups and interview verbatim. A grounded-theory approach guided analysis [19]. For our study, this analytic approach aimed to generate a theory or framework of ethical issues that could guide the development and content of the global-health ethics training sessions. Data were analyzed using constant comparative methodology [19]. In accordance with grounded theory, we did not propose any a priori hypotheses prior to data collection and we analyzed data inductively without reference to any predetermined theory [19]. The first level of coding involved “open coding” where we took raw segments of data and systematically reviewed them line-by-line, attributing specific descriptive labels or codes to the data [20]. Following this, we conducted “axial coding”, which involved the organization of codes identified during open coding into broader themes and categories based on observed relationships or common characteristics [21]. The final part of analysis involved “selective coding” whereby the discrete categories and themes we had identified were further refined into two overarching categories that integrated all of the other categories identified [19]. At least two reviewers independently analyzed the data and participated in all coding. Once each phase of coding had been completed, coders met and discussed the various codes and coding decisions, to determine agreement and disagreement. All discrepancies were resolved by negotiated consensus [22]. Data was analyzed in NVivo 9 (QSR International Pty Ltd., Doncaster, Australia).

## 3. Results

### 3.1. Participants

Eighteen global-health experts participated in three focus groups and an interview. Of the 18, nine were from the field of pharmacy, six from medicine (including an anthropologist and psychiatrist affiliated with the UCSF School of Medicine) and three from nursing. Eleven of the participants were female and seven male.

### 3.2. Key Ethical Themes and Topics

**Figure 1:**
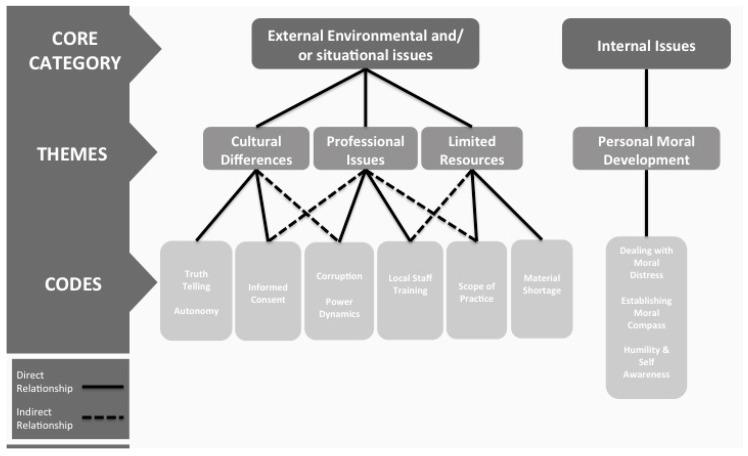
A summary of the ethical dilemmas (codes).

Participants provided 11 examples of ethical dilemmas associated with global-health outreach work that they had experienced and that they suggested trainees could also potentially encounter. These 11 ethical dilemmas (codes) fell into four major themes relating to: cultural differences, professional issues, limited resources, and personal moral development. Three of these themes (cultural differences, professional issues, limited resources) are categorized under the core category of external issues. This relates to external environmental and/or situational issues that trainees are confronted with. The fourth theme, moral development, reflects internal issues faced by each trainee, in a unique and individual manner, and refers more to the development of a moral compass and the exercise of humility and self-awareness than any environmental or situational issues a trainee might be facing. A summary of the ethical dilemmas (codes) that are part of each theme and core category are shown in Figure 1. In addition, this figure also highlights potential indirect relationships between some ethical issues and other themes that could be considered when developing curriculum content.

#### 3.2.1. Theme 1: Cultural Differences (Codes Include Informed Consent, Truth-Telling, and Autonomy)

The most common sources of ethical dilemmas participants identified related to difficulties with truth-telling and establishing informed consent. Participants noted that the concept of informed consent in resource-limited settings was very different to what they were familiar with at home. In some areas, health workers were expected to refrain from delivering diagnoses that would cause too much emotional distress to a patient. One participant proposed that in such a case one might argue:

“[…] that the beneficent thing to do is to withhold that information if the family is saying, “No, he can’t handle this. […] In that case, culturally, beneficence outweighs autonomy in most scenarios.”.

(Medicine Physician)

Others noted that informed consent was often related to cultural differences.

“…informed consent is a big issue… it goes back to what the cultural practice has dictated for so many years”.

(Nursing)

While Pharmacists noted that one of the main lessons for trainees while overseas is to clearly understand any cultural differences from the beginning:

“It’s important to respect whoever’s practice you are interacting with”.

(Pharmacist)

In other places, informing the patients of even the slightest possibility of adverse reaction was likely to result in their refusal to participate in treatment.

“[Where I worked], if you ask the patient, they will say, ‘Absolutely not. You are telling me there’s a risk in the procedure? I don’t want it.’”.

(Medicine Physician)

Similarly, the concept of autonomy was perceived to have a very different interpretation in global health settings. In fact, some participants argued that the idea of autonomy as a paramount value in the health professions should in fact be abandoned in cultures where it carries no such importance.

“Places where, for example, the word, autonomy, it’s like you can’t come up with a good translation for it. There’s just no word for it!”.

(Anthropologist)

#### 3.2.2. Theme 2: Professional Issues: (Codes Include Power Dynamics, Extent of Training of Local Staff, Perceived Corruption)

Some participants voiced concerns regarding the troubling power dynamics at some destinations. In some instances, trainees who had a “Western education” were perceived as possessing superior knowledge regarding clinical issues. Occasionally, even local professionals who were more experienced than the participants themselves would defer to their opinions.

“[…] these were mostly physicians, and I was the only nurse there. […] So I said ‘I wonder if [the traditional vaccinations] would also facilitate HIV transmission.’ And somebody at the table said, ‘Okay, we’re going to make a policy that nobody can give traditional vaccinations!’”.

(Nurse)

Participants also discussed the impact of differences in training between trainees and local staff. Some participants reported that the relative lack of resources at many global-health sites can result in local professionals who have often undergone less in-depth training than what they would have required in the U.S., and that local staff consequently often expected the trainees to provide a level of care that was beyond their own scope of practice.

“The scope of practice is constantly changing in the global setting. […] Nurses in [resource limited country] now, are doing Cesarean sections, are the prescribers of anti-retroviral therapy” […].

(Nurse)

“So many of the countries I’ve worked in have so few actual pharmacists, but they still have to have people to provide medicine. So you may be working with someone who you feel hasn’t gone through as much formal training as you might expect in the U.S.”.

(Pharmacist)

Another professional issue that many participants discussed was their perception of direct or indirect exposure to corruption.

“We had a similar situation there where we were going to collaborate with the department of public health to do the initial survey. They basically demanded a bribe, which was not small”.

(Pharmacist)

“They basically wanted us to pay them a fee, which was quite a hefty fee, in order for us to utilize them as a site. And they were saying that fee was for the ethics committee”.

(Pharmacist)

#### 3.2.3. Theme 3: Limited Resources (Codes Include Scope of Practice, Material Shortage)

Another common ethical theme concerned the expectation that participants practice outside of their scope of training, often because they were perceived to be the only person in a position to prevent a great harm from happening to the patient. In critically understaffed locations, participants had to make difficult decisions between performing procedures they were not qualified for or else leave the patient to die.

“We knew that likely this patient would die without a pericardiocentesis. […] Theoretically, we could have done it, and there was nowhere else to send the patient. But we decided that, because we had never done one, that we shouldn’t risk it. And the patient died overnight”.

(Medicine Physician)

Nurses also acknowledged the changing clinical practice challenges in resource-limited settings:

“The scope of practice is constantly changing in the global setting. So the scope of practice for nurses is constantly changing”.

(Nursing)

Participants discussed other cases where material shortages brought up additional dilemmas and caused distress to the participants.

“So in a limited-resource environment, even though it is something that can be reversed as easily as getting blood, [it] is sometimes delayed and people die because there is no blood or they’re trying to save it for a different case”.

(Pharmacist)

“I didn’t even know how to practice in that setting. There are no supplies. There are no blood pressure cuffs. There are no gloves. I couldn’t walk in there and do anything at the hospital”.

(Nursing)

“When I was on the wards in [host site] in January, there were a number of [U.S. Medical School] medical students and residents there and they all had on facemasks and they were the only ones on the ward. None of the other staff were wearing masks. And I was standing there with two medical students from somewhere else and thinking to ourselves, what should we do? …The wards are filled with HIV/TB patients, who are coughing”.

(Medicine Physician)

#### 3.2.4. Theme 4: Personal Moral Development: (Codes Include Dealing with Moral Distress, Establishing a Moral Compass, Humility and Self-Awareness)

Most ethical issues and dilemmas described so far caused significant moral distress, especially in inexperienced trainees. Moral distress in these examples refers to when an individual knows the correct and appropriate thing to do however institutional constraints make it difficult for them to pursue this action [23] Some thoughts offered by the focus group participants on this subject were:

“That’s what we’re not trained to deal with either: how that’s going to impact us emotionally and what’s our role in all that?”.

(Nurse)

“It may not be sustainable once we walk out the door, so do we save the person’s life at that moment or do we just continue to try and train people there about how to care for people? It’s upsetting”.

(Nurse)

“I think that the long-term consequences of that, mental health and other consequences for our students in particular, are really bad”.

(Medicine Physician)

Some participants stated that moral distress, while unpleasant, may actually assist the trainees in their personal development of obtaining a moral compass, as well as humility and self-awareness:

“If you consistently have moral distress even though you are doing the things that all the principles tell you […] part of what happens in ethical practice is figuring out what resonates with who you are as a human being. […] The idea is that you eventually develop this compass of practice.”.

(Medicine Physician)

“…what makes you a great medical student in the States often makes you slightly obnoxious abroad. Try to have humility and awareness”.

(Medicine Physician)

Participants agreed that the important thing in all morally distressing situations is that global health trainees have a reliable moral and personal guidance that they can rely on. They need to know how and where to seek help when faced with difficult moral and ethical dilemmas.

“The trainees that I work with are mainly integrated in the programs and they’re getting certain skills, and they know they are going to work in the field long term. So I think they feel a little less distressed because they feel a little empowered.”.

(Medicine Physician)

## 4. Discussion

This study has identified a diverse range of ethical issues from a multi-professional group of Medicine, Nursing and Pharmacy faculty who described ethical challenges that they had experienced and believed that trainees may also encounter when they are working in resource-limited settings. Faculty noted that many trainees on placements could struggle with ethical dilemmas on both professional and personal levels, and that training in this area prior to embarking on their global-health missions would be beneficial. Even returning trainees who have been exposed to these dilemmas on their previous missions would likely benefit from discussion and counseling on how to address ethical challenges.

These findings are important because, although ethical themes in global-health work have been described in the past, for example the broader issues of social justice, solidarity, humility, and introspection [11], the specific ethical issues described can further inform existing, or future, in-person, online or simulation-based global health ethics training. Our findings resonate with other work investigating the same topic. For example, challenges with informed consent, truth telling, scope of practice and local staff training have been described elsewhere [6,3,24]. To our knowledge the ethical issues identified in this study such as perceived corruption, and many of the issues related to our final theme, personal moral development, have not been widely discussed in the context of ethical challenges during global health placements. More broadly, despite some of the topics identified during our qualitative study being reported elsewhere, the specific scenarios we describe are largely new and can be utilized as case studies or vignettes during global health ethics training. For example, Stanford and John Hopkins online global health ethics training currently present a case study under the topic “shifting resources” that describes how local resources can inappropriately be diverted to support trainees on their placement [25]. Related to resources are the “material shortages” examples described in our current study where trainees may have to make difficult decisions about rationing limited resources.

Many participants identified cultural differences as a source of ethical dilemmas in their global-health experience. The effects of different traditions, religions, and cultural norms were noticeable in their dealings with the local staff and patients, and they often affected the participants’ relationships with their patients and colleagues and placed them in genuine ethical dilemmas. For example, participants reported cases that compelled them to reconsider their duty to inform the patients of the true nature of their affliction and the circumstances of the proposed treatment. Given the discussion across all professional groups of how cultural differences can create challenges for trainees, it is not surprising to find that teaching around this topic is found in most existing global health ethics training programs [12,13,16].

Another repeated concern involved the expectation that trainees perform outside of their scope of practice due to insufficient workforce at local sites. Elansary et al. describe this phenomenon as “clinical limits” in their series of case studies highlighting ethical dilemmas in global health electives [24]. Many global-health settings are severely understaffed and it is therefore often expected that the visiting staff will perform the tasks for which they are insufficiently trained, since the only other alternative is for patients to suffer severe clinical consequences, even death. Sugar and Crump add that host institutions often have inflated ideas about the skill level of trainees on short-term placements adding to this difficult situation [9]. Radstone supports this view in their study reporting that 80% of local health workers in the Solomon Islands did not understand the clinical limits of visiting trainees [26]. Scope of practice issues place visiting trainees in extremely difficult ethical dilemmas, where the norms they have been taught in school challenged their ethical intuitions in unfamiliar situations.

Participants in our study reported that their global health experiences made them reconsider the lessons they have previously learned about the absolute importance of autonomy, informed consent, and truth-telling. They further discussed how such occurrences lead them to re-examine their ethical notions and values, and made them reflect on moral issues they were previously unaware of. These issues also facilitated their personal moral development, as well as prompted them to start building a theoretical approach regarding global moral issues.

When categorizing the ethical issues into four themes, we recognized that they further align into two overarching core categories: those themes that referred to external factors faced by trainees (cultural issues, professional issues, and limited resources) and the theme which referred to internal factors faced by trainees on a personal level (personal moral development). This was an interesting finding—the divide between external and internal factors has not been identified before to our knowledge and has educational implications since external factors are much easier to present as case vignettes or to simulate, whereas internal factors are more subjective and harder to elicit for each trainee, especially in the short period of time during which most ethics training transpire.

While our framework (Figure 1) generalizes many potential ethical global health scenarios and we support trainees maintaining a nuanced approach to each situation they encounter, our findings are of importance for future global health-ethics training development, and we propose that the categorization represented in our framework (Figure 1) should be consulted when preparing global health trainees for their fieldwork. In fact, many of the ethical issues identified are not limited to practicing abroad and may also reflect challenges trainees face in pluralistic communities in their home countries. These themes can be used to inform existing and proposed global-health training and perhaps more broadly health professional curriculum design, regardless of the approach utilized. Further, the multi-professional sample in our study suggests that the material can be used to help educate trainees in different health sciences.

Our study has certain limitations; this is a single center study with a small number of participants. Given the limited number of interviews and focus groups, we were not able to determine if thematic saturation was achieved. Investigation of a larger group of participants could contribute additional experiences that would affect our categorization of ethical topics and themes. Our findings also do not illustrate ethical challenges in global health settings based on inter-professionalism; they represent the perspectives of different health science professionals. A further challenge is that individual themes such as those categorized under moral distress are challenging to replicate in experiential learning activities. We also acknowledge that the results of our study are based on the perspectives of U.S.-based health professionals, whose interpretation of ethical issues will likely be different to those local providers living and working in the resource-constrained setting. Further, this study did not obtain the perspectives of trainees. Future work should include the perspectives of both these stakeholders.

## 5. Conclusions

The results of this study have identified two core overarching educational goals of a pre-departure global-health curricula. This includes exposing trainees to external environmental and/or situational ethical dilemmas related to cultural differences, professional differences, and limited resources, as well as providing them with skills necessary to develop an internal moral compass and exercise of humility and self-awareness. The findings from our study will be useful for curriculum content and vignettes for global-health ethics training.
